# ACUTE GENERALIZED EXANTHEMATOUS PUSTULOSIS: AN UNUSUAL SIDE EFFECT OF MEROPENEM

**DOI:** 10.4103/0019-5154.62759

**Published:** 2010

**Authors:** Mohammed Hanafy Khalel, Salhamooud Abdel Fattah Saleh, Abdel-Hamid F El-Gamal, Nabeel Najem

**Affiliations:** *From the Dermatology Department, Adan Hospital, State of Kuwait, Kuwait*; 1 *From the Internal Medicine Department, Adan Hospital, State of Kuwait.*

**Keywords:** *Post hepatitic C*, *pustular eruption*, *meropenem*

## Abstract

A male patient was hospitalized as a case of pneumonia. He was diabetic, hypertensive and post Hepatitis “C” “H-C”. He reported skin eruption following administration of meropenem. Skin biopsy revealed acute generalized exanthematous pustulosis. To elucidate this side effect, we conducted a literature search - this is the second case induced by meropenem. The diagnosis was made after excluding all other possible causes. Dermatologists and clinicians must be aware of this an unusual side effect.

## Introduction

Acute generalized exanthematous pustulosis (AGEP) is a rare severe cutaneous reaction pattern which, in majority of the cases, is related to medication.[1] Internal organ involvement is relatively rare and the mortality rate is approximately 5%.[2] The etiopathogenesis of AGEP is still obscure. Viral causes have been reported by some authors.[3] Pharmaceutical drug intake is considered the main causative agent in 87% of the cases, among which the most important are antibiotics, especially beta-lactam and macrolides.[4]

AGEP, in some cases, may manifest initially as psoriasis. This is disregarded if there is no recurrence of the psoriasiform lesion within the two years following the clinical presentation.[5] We report the second AGEP case induced by meropenem; the first was reported in 2003.[6]

## Case Report Induced by Meropenem

**Figure 1:**
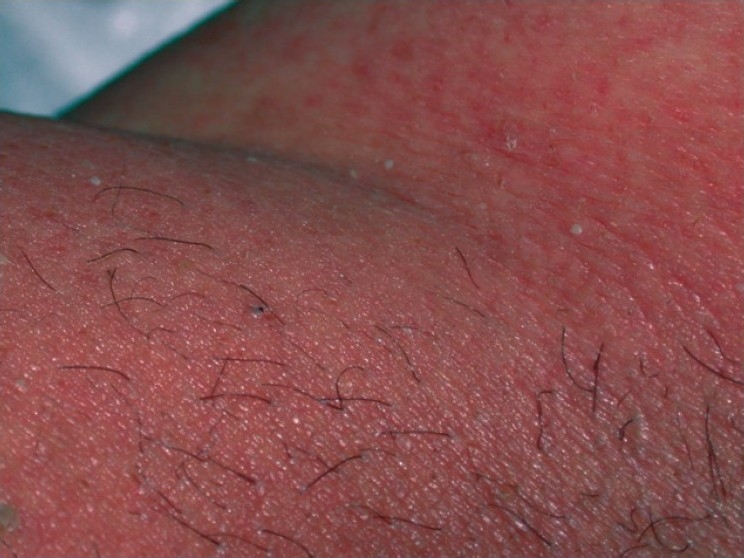
small subcorneal pustules on erythematous patches

A 45-year-old male patient was admitted to the hospital with chest infection and fever. He had a history of post hepatitis C, chronic renal insufficiency, insulin requiring diabetes mellitus, hypertension and dilated cardiomyopathy. Chest X-ray revealed right basal pneumonia. The patient who was administered meropenem for chest infection reported an exanthematous skin eruption within 24 hours. Dermatological examination revealed bilateral symmetrical erythematous confluent patches on trunk and extremities with close standing small, subcorneal pustules [Figure 1]. Purpuric lesion was observed on lower trunk and lower extremities and upper extremities; pustule was present on lower lip and ventral surface of tongue.

Investigations showed leukocytosis. Bacterial and fungal cultures of the pustular lesions were negative. Skin biopsy revealed, subcorneal neutrophilic pustules, the upper dermis shows extravasation of RBCs, admixed with neutrophils and perivascular infiltrate of lymphocytes, histiocytes, neutrophils and eosinophils. Mild dermal edema was also noted but fibrinoid necrosis was not detected.

A diagnosis of AGEP due to meropenem was done. We stopped meropenem, and changed to erythromycin and ciprofloxacin for chest infection in addition to prior treatment for diabetes mellitus, hypertension, cardiac problem, and treatment for renal problems. In addition, prednisolone tablet 60mg/day was used, followed by tapering. The condition of AGEP was controlled and the skin returned to normal condition within 12 days.

## Discussion

AGEP is a clinical reaction pattern induced in more than 90% of cases by systemic drugs. It is a rare manifestation of an adverse drug reaction, mostly induced by anti-infective drugs. The Drug Eruption Reference Manual lists 63 drugs reported to cause AGEP.[7] AGEP is defined by rapid onset following the introduction of the drug (Less than 24 hours) as in our case. Clinically, it is characterized by polymorphism of eruption, single episode, absence of arthritis and frequent administration of drugs.[8] The involution of the condition is slower, taking between 10 and 14 days,[1] as in our case in which complete recovery occurred within 12 days.

Clinically, the lesions of AGEP started on the face and within a few hours spread to the trunk and limbs, or started to arise in intertriginous areas. After that there was annular desquamation for a few days, possibly accompanied by polymorphic lesions, especially purpuric lesions on the legs and feet. The mucous membranes are affected in 25% of the cases. As described in our case the eruption appeared within 24 hours of administration of the drug associated with fever. There was regression of the condition after discontinuation of medication and introduction of corticosteroid treatment. Histologically, the differential diagnosis from pustular psoriasis is made by absence of hyperplasia of the epidermis and papilloacanthosis.[9]
